# Hidden Costs of the Green Transition: A Bioethical Analysis of Occupational Hazards in Lithium-Ion Battery Supply Chains

**DOI:** 10.7759/cureus.117670

**Published:** 2026-10-09

**Authors:** Marta Dias, Patrícia P Almeida

**Affiliations:** 1 Occupational Medicine, Hospitais da Universidade de Coimbra, Coimbra, PRT; 2 Occupational Health, Unidade Local de Saúde do Médio Tejo, Torres Novas, PRT

**Keywords:** bioethics, environmental justice, lithium-ion batteries, mining, occupational exposure, occupational health, supply chain, sustainability

## Abstract

The global transition toward low-carbon energy has made lithium-ion batteries essential to electric mobility and renewable energy storage. Yet the environmental benefits of this transition can obscure an important ethical contradiction: workers involved in extracting and processing lithium, cobalt, nickel, and manganese may face substantial occupational hazards. Exposure to respirable dust, toxic metals, and chemical agents occurs across battery mineral supply chains, often in settings where regulatory protection and occupational health surveillance are limited. A transition that reduces carbon emissions while tolerating preventable occupational harm cannot be considered fully sustainable. From a bioethical perspective, such a transition must uphold the principles of justice, non-maleficence, and respect for human dignity. Occupational health should therefore be treated as a core indicator of sustainability. Governments, industries, investors, and consumers share responsibility for ensuring that decarbonization does not depend on transferring health risks to vulnerable workers. A genuinely just energy transition must protect both the planet and the people whose labor makes that transition possible.

## Editorial

Lithium-ion batteries have become one of the defining technologies of the green energy transition. They power electric vehicles, support renewable energy storage, and contribute to strategies aimed at reducing dependence on fossil fuels [1,2]. Their expansion is therefore commonly presented as evidence that technological innovation can reconcile economic development with environmental responsibility [1]. However, this optimistic narrative deserves closer ethical scrutiny. The question is not simply whether a technology reduces carbon emissions, but also who bears the human cost of producing it.

The extraction and processing of lithium, cobalt, nickel, and manganese illustrate this dilemma. Workers involved in these activities can be exposed to respirable crystalline silica, metal-containing dust, and other hazardous substances associated with respiratory, neurological, cardiovascular, and other adverse health effects [1-3]. Such risks are particularly concerning when they intersect with informal employment, inadequate personal protective equipment, limited occupational surveillance, and weak regulatory enforcement.

Cobalt extraction in the Democratic Republic of the Congo is perhaps the most visible example of this problem. Artisanal and small-scale mining can occur under precarious working conditions, demonstrating how the benefits and burdens of technological progress may be distributed unequally. Consumers thousands of kilometers away benefit from electric mobility and cleaner technologies, while workers and communities at the beginning of the supply chain may bear occupational and social costs that remain largely invisible to the final consumer [1,2].

However, it would be misleading to frame this exclusively as a problem affecting low-income countries. Occupational exposure also occurs within highly regulated mining industries [1,4]. Evidence concerning respirable dust and crystalline silica among lithium mine workers in Western Australia, for example, demonstrates that legislation alone does not eliminate occupational hazards [4]. Similarly, emerging evidence from lithium-ion battery material production raises concerns about exposure to particles containing nickel, cobalt, and manganese [3]. The issue is therefore not simply where minerals are extracted or processed, but whether occupational health protection is developing at the same pace as demand for these materials.

This raises a fundamental question: can an energy transition be described as “green” if preventable harm to workers remains embedded within its supply chains?

From a bioethical perspective, the answer should be no. Environmental sustainability cannot be separated from justice, non-maleficence, and respect for human dignity. Carbon reduction is unquestionably important, but environmental benefits cannot provide ethical justification for avoidable occupational disease. When the benefits of decarbonization accrue primarily to some populations while occupational risks are disproportionately transferred to others, the transition risks reproducing the same structural inequalities that sustainable development is intended to overcome [1,2].

This perspective also changes how occupational risk should be managed. Personal protective equipment remains necessary in many circumstances, but it should not become the primary answer to hazards that could be eliminated or controlled upstream [1,4]. The hierarchy of risk control reminds us that eliminating hazards, substituting safer materials or processes, and implementing effective engineering controls are preferable to placing responsibility predominantly on individual workers [1,4]. Automation, enclosed processing systems, ventilation, dust suppression and exposure monitoring should therefore be considered part of the technological innovation required for the energy transition, rather than additional costs external to it [1,3,4].

Transparency is equally important. A battery should not be considered sustainable solely because of its carbon performance. Responsible sourcing should also account for the conditions under which its constituent minerals were extracted and processed. Traceability, independent auditing, occupational exposure monitoring, and meaningful human rights due diligence can help make hidden risks visible [1,2]. Yet voluntary corporate commitments are insufficient when they lack enforceability. Ethical supply-chain governance requires accountability, not merely sustainability branding. The European Union Battery Regulation establishes due diligence requirements addressing social and environmental risks associated with raw materials used in battery production [5]. This regulatory framework represents an important step toward greater transparency and accountability; however, its effectiveness ultimately depends on robust implementation, independent verification, and enforcement.

There is also a danger in assuming that technological innovation alone will solve these problems. Automation can remove workers from hazardous environments, but it can also eliminate livelihoods or create new inequalities when introduced without appropriate training and social protection [1-3]. A just transition therefore requires worker participation. Those exposed to occupational hazards should not simply be recipients of safety policies; they should have a meaningful voice in identifying risks, designing preventive strategies, and evaluating whether protective measures actually work.

Responsibility cannot rest exclusively with mining companies or producing countries. Battery supply chains are transnational, and so is the ethical responsibility associated with them [1,2]. Governments must establish and enforce appropriate occupational standards. Manufacturers should demand verifiable safety conditions throughout their supply chains. Investors should treat occupational health as a material component of environmental, social, and governance performance. Importing countries should avoid benefiting from regulatory disparities that allow hazardous production to migrate toward less protected populations. Consumers, although more distant from production decisions, should also recognize that a low-carbon product is not automatically an ethically sustainable product.

The concept of a “just transition” must consequently extend beyond protecting workers whose jobs may disappear as fossil-fuel industries decline. It must also encompass the workers whose labor makes renewable technologies possible [1,2]. Otherwise, society risks replacing one form of environmental injustice with another.

The success of the green transition should therefore be measured not only in tonnes of carbon avoided, electric vehicles sold, or renewable capacity installed, but also by whether workers can produce the materials required for that transition without sacrificing their health. Occupational diseases are not an inevitable price of decarbonization [1,3,4].

Climate action and occupational health should not be competing objectives. They are components of the same sustainability agenda. A transition capable of protecting the climate while disregarding the health and dignity of the people who enable it remains ethically incomplete. The challenge ahead is therefore not merely to build a low-carbon economy, but to ensure that the path toward it is also safe, equitable, and worthy of being called a just transition.
